# HY5 Contributes to Light-Regulated Root System Architecture Under a Root-Covered Culture System

**DOI:** 10.3389/fpls.2019.01490

**Published:** 2019-11-28

**Authors:** Yonghong Zhang, Chunfei Wang, Hui Xu, Xiong Shi, Weibo Zhen, Zhubing Hu, Ji Huang, Yan Zheng, Ping Huang, Kun-Xiao Zhang, Xiao Xiao, Xincai Hao, Xuanbin Wang, Chao Zhou, Guodong Wang, Chen Li, Lanlan Zheng

**Affiliations:** ^1^Laboratory of Medicinal Plant, Institute of Basic Medical Sciences, School of Basic Medicine, Biomedical Research Institute, Hubei Key Laboratory of Wudang Local Chinese Medicine Research, Hubei Key Laboratory of Embryonic Stem Cell Research, Hubei University of Medicine, Shiyan, China; ^2^Center for Multi-omics Research, Key Laboratory of Plant Stress Biology, School of Life Sciences, Henan University, Kaifeng, China; ^3^College of Life Sciences, Nanjing Agricultural University, Nanjing, China; ^4^National Engineering Laboratory for Resource Development of Endangered Crude Drugs in Northwest China, Key Laboratory of Medicinal Resources and Natural Pharmaceutical Chemistry, Ministry of Education, College of Life Sciences, Shaanxi Normal University, Xi'an, China; ^5^Department of Biological Science, Florida State University, Tallahassee, FL, United States; ^6^Jiangsu Key Laboratory of Marine Biological Resources and Environment, Jiangsu Key Laboratory of Marine Pharmaceutical Compound Screening, Co-Innovation Center of Jiangsu Marine Bio-industry Technology, Jiangsu Ocean University, Lianyungang, China; ^7^Key Laboratory of Three Gorges Regional Plant Genetics & Germplasm Enhancement (CTGU)/Biotechnology Research Center, China Three Gorges University, Yichang, China

**Keywords:** *Arabidopsis*, HY5, light, root photomorphogenesis, root system architecture, soil, transcriptome

## Abstract

Light is essential for plant organogenesis and development. Light-regulated shoot morphogenesis has been extensively studied; however, the mechanisms by which plant roots perceive and respond to aboveground light are largely unknown, particularly because the roots of most terrestrial plants are usually located underground in darkness. To mimic natural root growth conditions, we developed a root-covered system (RCS) in which the shoots were illuminated and the plant roots could be either exposed to light or cultivated in darkness. Using the RCS, we observed that root growth of wild-type plants was significantly promoted when the roots were in darkness, whereas it was inhibited by direct light exposure. This growth change seems to be regulated by ELONGATED HYPOCOTYL 5 (HY5), a master regulator of photomorphogenesis. Light was found to regulate *HY5* expression in the roots, while a HY5 deficiency partially abolished the inhibition of growth in roots directly exposed to light, suggesting that *HY5* expression is induced by direct light exposure and inhibits root growth. However, no differences in *HY5* expression were observed between illuminated and dark-grown *cop1* roots, indicating that HY5 may be regulated by COP1-mediated proteasome degradation. We confirmed the crucial role of HY5 in regulating root development in response to light under soil-grown conditions. A transcriptomic analysis revealed that light controls the expression of numerous genes involved in phytohormone signaling, stress adaptation, and metabolic processes in a HY5-dependent manner. In combination with the results of the flavonol quantification and exogenous quercetin application, these findings suggested that HY5 regulates the root response to light through a complex network that integrates flavonol biosynthesis and reactive oxygen species signaling. Collectively, our results indicate that HY5 is a master regulator of root photomorphogenesis.

## Introduction

Root system architecture (RSA) is vital for plant fitness, crop performance, and plant productivity, and its development is regulated by both genetic components and environmental factors (Jansen et al., 2013; Rogers and Benfey, 2015; Li et al., 2018a; Xiao et al., 2018). Light has largely been neglected as an environmental factor controlling RSA since the root systems of most terrestrial plants remain underground in darkness (Osmont et al., 2007; Bellini et al., 2014). Intriguingly, the genes encoding photoreceptors are expressed in the roots, enabling them to detect ambient light and trigger downstream responses that mediate root tropism and elongation in the soil (Galen et al., 2007; Salisbury et al., 2007; Mo et al., 2015; Van Gelderen et al., 2018b). For instance, red light was found to activate the phytochrome A (phyA)- and phyB-mediated signal transduction pathways in the root and dramatically inhibit root elongation in etiolated seedlings (Correll and Kiss, 2005). Roots exposed to direct illumination immediately generate a strong burst of reactive oxygen species (ROS), leading to the redistribution of auxin (Yokawa et al., 2011); however, the mechanisms by which light regulates RSA development have not been fully elucidated.

Light-mediated shoot development (i.e., shoot photomorphogenesis) has been much more extensively studied than light-mediated root development (root photomorphogenesis) (Briggs and Lin, 2012; Gommers and Monte, 2018; Van Gelderen et al., 2018b). Numerous downstream components of the photoreceptor signaling pathways have been characterized (Arsovski et al., 2012; Li et al., 2012). The RING E3 ubiquitin ligase CONSTITUTIVE PHOTOMORPHOGENIC 1 (COP1), a major integrator of light responses, ubiquitinates two downstream transcription factors, ELONGATED HYPOCOTYL 5 (*HY5*) and *HY5* HOMOLOG (HYH), to mediate their degradation (Ang et al., 1998; Osterlund et al., 2000; Holm et al., 2002). HY5 is a positive regulator of photomorphogenesis (Lee et al., 2007; Li et al., 2010; Wang et al., 2019). The loss-of-function *hy5* mutant produces elongated hypocotyls when grown under constant light, while the *hyh* mutant exhibits no obvious aberrant phenotype (Oyama et al., 1997; Holm et al., 2002; Sibout et al., 2006). The expression levels of *HY5* and *HYH* are upregulated by light in the seedlings (Osterlund et al., 2000; Holm et al., 2002; Singh et al., 2012).

Generally, root growth and development in response to light are controlled by both shoot-derived and root-autonomous signals. Among the shoot-derived signals, phytohormones and peptides are regarded as important players (Bhalerao et al., 2002; Li et al., 2018b; Qu et al., 2019); for example, regardless of whether roots were exposed to light or darkness, the illumination of shoots efficiently promoted root growth by inducing the expression of *DWF4*, which encodes a brassinosteroid (BR) biosynthesis enzyme, leading to an increased accumulation of BRs in the roots (Sakaguchi and Watanabe, 2017). The COP1-mediated shoot-to-root transport of auxin is also involved in adapting root growth to the ambient light conditions (Sassi et al., 2012), with the downstream transcription factor HY5 regulating auxin accumulation in the roots by modulating the intracellular distribution of the auxin transporter PIN-FORMED2 (PIN2) (Laxmi et al., 2008).

Besides phytohormones, HY5 acts as a mobile shoot-to-root signal that promotes nitrate uptake in roots in response to light (Chen et al., 2016). In addition, aboveground light is directly transmitted through the stem to activate phytochrome B in the roots, thereby triggering HY5-mediated light responses (Lee et al., 2016). Root-autonomous signals might also be partially generated through metabolic processes; for example, Silva-Navas et al. (2016) reported that the accumulation of flavonols in the root transition zone mediates the root response to light and therefore proposed that flavonols function as local positional signals (Silva-Navas et al., 2016).

In the laboratory, seedlings are often grown on transparent Petri dishes, exposing the roots to light, despite roots naturally growing in the soil and avoiding light. To achieve a better understanding of RSA development under natural light conditions, researchers have developed different strategies to culture shoots and roots in separate light conditions (Xu et al., 2013; Yokawa et al., 2014; Silva-Navas et al., 2015; Sakaguchi and Watanabe, 2017). In this study, we developed a root-covered system (RCS) to investigate the mechanism by which light regulates root development. We found that the RSA is enhanced in plants grown in darkness compared to roots exposed to light, which was partially dependent on HY5. Furthermore, a transcriptomic analysis revealed that light-mediated regulation of root development might be based on a complex network involving phytohormones, stress signaling, and biosynthetic and metabolic processes and that some of these regulatory pathways are dependent on HY5.

## Materials and Methods

### Plant Materials and Growth Conditions

All *Arabidopsis thaliana* mutants used in this study were originally generated in the Columbia-0 (Col-0) background. The *hy5* (SALK_056405), *hyh* (WISCDSLOX253D10), *hy5 hyh*, and *cop1-6* mutants were described previously (Sassi et al., 2012; Zhang et al., 2017). The seeds were germinated on half-strength Murashige and Skoog (1/2 MS) agar plates or on soil (covered with a thin layer of sand to shield the roots from light) and were incubated at 22°C under continuous white light (120 µmol m^−2^ s^−1^) or in darkness (covered with foil) in a plant growth chamber for 7 days, or as indicated.

**Figure 1:**
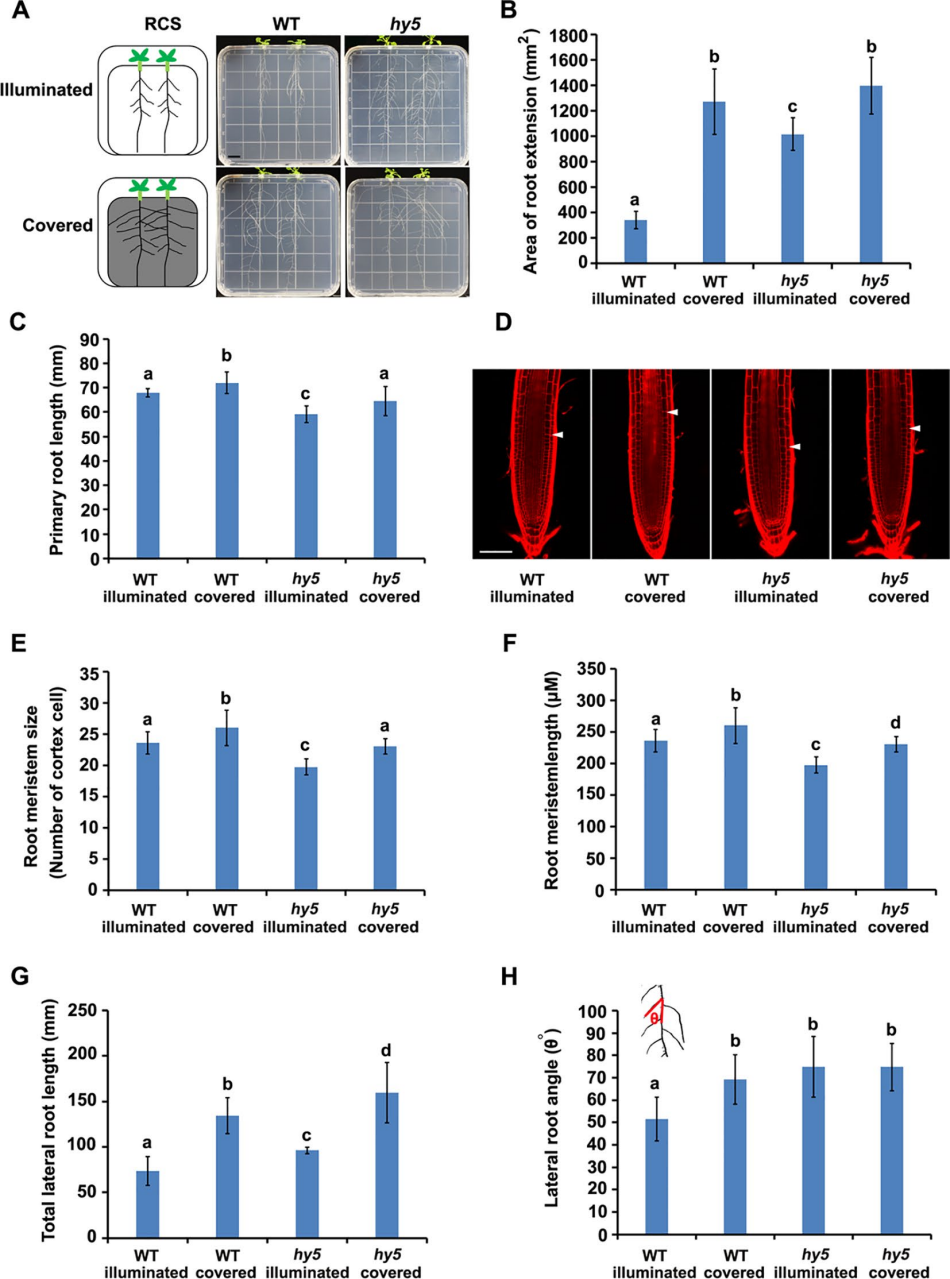
Phenotypes of root system architectures (RSA) of wild-type (WT) plants and *hy5* mutants under root-covered system (RCS). **(A)** Representative pictures of the RSA phenotypes of 15-day-old WT and *hy5* plants. *Scale bar* represents 10 mm. **(B)** Quantification of the root extension area (in square millimeters) of WT-illuminated, WT-covered, *hy5*-illuminated, and *hy5*-covered. **(C)** Quantification of the primary root lengths of 9-day-old WT and *hy5* plants. **(D**–**F)** Root meristem phenotypes of 9-day-old WT and *hy5* plants presented as representative confocal images **(D)**, meristem sizes (measured as the number of cortex cells) **(E)**, and the length of root meristems **(F)**. *Scale bar* in **(D)** represents 50 μm. **(G)** Total lateral root lengths of 9-day-old WT and *hy5* plants. **(H)** Average lateral root angles of 9-day-old WT and *hy5* plants. The *inset* on the *upper left* denotes how the angles were measured. *Error bars* represent the standard deviation (SD) of biological triplicates (*n* = 6). Different letters indicate significantly different values at *P* < 0.05 (pairwise Student's *t* test).

To accomplish the covered-root culture condition, two pinholes were made on the top edge of a square plastic Petri dish (9 × 9 cm) containing 1/2 MS medium. Two 3-day-old etiolated seedlings were transferred into the corresponding pinholes using fine forceps, ensuring that the roots were placed on the medium inside the dish with the hypocotyls passing through the pinholes, leaving the cotyledons outside the dish. The Petri dishes were subsequently either covered with double layers of foil to conceal the root but not the shoot (roots are covered) or sealed without foil (roots are illuminated). The dishes were then placed into larger dishes (13 × 13 cm) to create a relatively sterile environment and the seedlings were grown for 12 additional days, or as indicated (**Figure S1** and **Figure 1**).

For the light–dark transition experiments, the seedlings were grown in the soil under continuous light or darkness for 5 days, after which they were transferred to the opposite condition for 24 h. At least three biological replicates were performed.

For quercetin treatment, 3-day-old etiolated seedlings were transferred onto a solid 1/2 MS medium containing either a mock control or 50 µM quercetin for the indicated number of days.

### Expression and Phenotypic Analyses

β-glucuronidase (GUS) staining was performed as described previously (Zhang et al., 2017; Liu et al., 2019a; Zhang et al., 2019). Briefly, 7-day-old seedlings were incubated in the assay buffer [50 mM sodium phosphate (pH 7.0), 10 mM EDTA, 1 mM potassium ferricyanide, 1 mM potassium ferrocyanide, 0.1% Triton X-100, 20% methanol, and 0.5 mg ml^–1^ X-Gluc A] at 37°C until sufficient staining was observed. The GUS activity was analyzed using a Nikon 80i microscope with Nomarski interference contrast optics.

The confocal imaging was performed using a Leica TCS SP2 microscope. Propidium iodide (PI; Sigma-Aldrich; 10 µg ml^–1^ dissolved in water) was used to counterstain the root cells. The fluorescence signals of green fluorescent protein (GFP) and PI were excited using an argon ion laser. The excitation and emission wavelengths for GFP were 488 and 510 nm, respectively. The excitation and emission wavelengths for PI were 561 and 630 nm, respectively.

To analyze the root phenotypes, the seedlings were imaged with a Canon 40D camera. The areas of root extension were indicated by tracking all the lateral roots (Ristova et al., 2013) and measuring their area using ImageJ with the SmartRoot plug-in (Lobet et al., 2011). Quantitative analyses of the root apical meristem (RAM) and lateral root angles were performed as previously described (Li et al., 2017a; Zhang et al., 2017). Statistical analyses (pairwise two-tailed Student's *t* tests) were performed using Microsoft Excel. Each experiment was performed in three biological replicates.

### RNA Extraction and Quantitative Reverse Transcription PCR

Total RNA was isolated from the roots using Tranzol (Transgene), in accordance with the manufacturer's protocol. The RNA purity was checked using a Nanodrop ND-1000 spectrophotometer (Thermo Fisher Scientific). Only samples with absorbance ratios of OD_260/280_ ≥ 1.8 and OD_260/230_ ≥ 1.8 were selected for further use. The cDNA was prepared using a PrimeScript RT Reagent Kit (RR047A; Takara Bio). The relative expression levels were determined by performing a quantitative reverse transcription PCR (qRT-PCR) analysis using an ABI 7500 Real-Time PCR System (Thermo Fisher Scientific) or a ViiA 7 Real-Time PCR System (Life Technologies). The PCR thermocycling protocol was as follows: 95°C for 5 min, followed by 40 cycles of 95°C for 15 s and 60°C for 1 min. After the amplification cycles, dissociation analyses were performed to confirm the validity of the target (Guo et al., 2018; Jiao et al., 2019; Li et al., 2019c) Three biological replicates and three qRT-PCR technical replicates were performed for each sample. *EF1α* (AT5G60390) was used as the reference gene for normalization. The primer sequences used for the qRT-PCR analyses are listed in **Table S1**.

### RNA Sequencing and Data Analyses

The roots of 15-day-old wild-type (WT) and *hy5* seedlings grown in illuminated or covered conditions were collected, immediately frozen in liquid nitrogen, and stored at −80°C before an RNA extraction. Three independent biological replicates were collected for each genotype and growth condition, and the total RNA was extracted as described above. After purification, the integrity of the RNA was verified using the RNA 6000 Nano Labchip Kit on an Agilent 2100 Bioanalyzer (Agilent Technologies), following the manufacturer's protocol. The enrichment of the mRNA from the total RNA, the cDNA synthesis, and the construction of the cDNA library were performed by the Beijing Genome Institute (BGI, China). An Illumina HiSeq 2000 sequencing system was used to sequence the libraries. After removing the sequences containing more than 5% unknown bases or more than 30% nucleotides with a sequence quality value below 10, the clean reads were aligned to the *Arabidopsis* genome using HISAT (Kim et al., 2015). The expression level of each gene was normalized as the number of clean reads per kilobase of exon per million mapped reads (RPKM) (Mortazavi et al., 2008). The method DESeq2 was used to select differentially expressed transcripts, while a false discovery rate (FDR) < 0.05 was further used to estimate the correction for false positive and false negative errors (Love et al., 2014). A heat map of differential expression was generated using custom R scripts based on Bioconductor packages (Gentleman et al., 2004), and the Venn diagrams were produced using the online software jvenn (Bardou et al., 2014). Gene Ontology (GO) annotations and GO singular enrichment analysis (SEA) were performed using goseq R Bioconductor package (Young et al., 2010). An FDR cutoff of 0.05 was used to determine the enriched GO pathways.

### ROS Level Determination

Fluorescein diacetate (FDA) was used to detect the ROS level in the roots, following the method described previously by Silva-Navas et al. (2016), with a slight modification. Briefly, the roots were submerged in a solution containing 0.01% Triton X-100 and 0.83 mg ml^–1^ fluorescein diacetate for 10 min. After being washed in deionized water for 2 min, the root tips were mounted on slides and imaged using a Nikon/A1+N-SIM confocal laser scanning microscope. The excitation and emission wavelengths for FDA were 488 and 520 nm, respectively. Three biological replicates were performed.

### Extraction and Determination of Flavonols

The root samples were homogenized in liquid nitrogen, and the flavonols were extracted in 80% acetone. The simultaneous rapid separation of the flavonoids was performed using high-performance liquid chromatography (HPLC) with 5-µm (150 × 4.6 mm) Bridge C18 columns. The linear gradient elution was performed using a mobile phase (0.1% formic acid and acetonitrile acidified with 0.1% formic acid) starting with 95% formic acid and ending with 5% formic acid over a total run time of 35 min. The flow rate of the mobile phase was 1 ml min^–1^. A 2998 PDA detector was used.

## Results

### HY5 Regulates RSA Development, as Revealed Using the RCS

In nature, plant roots grow in the soil and perceive little to no light (Galen et al., 2007; Sassi et al., 2012). For a long time, plant biologists have been growing *Arabidopsis* seedlings on transparent Petri dishes with the roots exposed to light, an approach we designated as a traditional culture system (TCS). Using a TCS, plant roots that would naturally grow in darkness were instead exposed to light (**Figures S1A, B**), which is controversial in the field of root research (Xu et al., 2013; Yokawa et al., 2013; Mo et al., 2015). To better understand the natural RSA of a plant, various approaches have been developed to mimic natural conditions (Xu et al., 2013; Yokawa et al., 2013; Silva-Navas et al., 2015; Lee et al., 2016; Sakaguchi and Watanabe, 2017). In this study, we developed a laboratory culture system called a root-covered system (RCS). Under the RCS, the plant shoots were exposed to light, while the exposure of the roots to light could be manually controlled by the presence or absence of a foil cover (**Figure S1** and **Figure 1**).

We examined the RSA of plants grown using the RCS. In WT seedlings, we observed a dramatic increase in the area of root extension of the seedlings grown with the foil cover (WT-covered) compared to those grown without the foil cover over the roots (WT-illuminated) (**Figures 1A, B**), demonstrating the regulatory role of light on root growth.

HY5 has been characterized as a key regulator of photomorphogenesis. To investigate whether HY5 is involved in the light-mediated regulation of RSA development, we grew the *hy5* loss-of-function mutant in the RCS under illuminated and covered conditions. Intriguingly, the area of root extension of the *hy5* mutants grown in the illuminated condition was dramatically higher than that of the WT illuminated seedlings (**Figures 1A, B**). Furthermore, the increase in root growth observed in the WT seedlings grown with the foil cover (WT-covered) relative to the illuminated condition was greater than that of the *hy5* seedlings (**Figures 1A, B**), with a 4.0-fold increase in the WT and a 1.3-fold increase in *hy5*. Thus, HY5 plays a role in regulating RSA in response to light.

A further analysis showed that both darkness and the *hy5* mutation increased the total lateral root length, lateral root angle, primary root length, and root hair lengths of the plants (**Figures 1C–E** and **Figure S2**). As root length is tightly correlated with root meristem activity (Sassi et al., 2012), we also analyzed the meristem size of the primary roots, revealing that darkness increased the size and length of the root meristem in both the WT and *hy5* plants (**Figures 1F–H**).

Auxin is the main regulator of lateral root development (Audenaert et al., 2013). To test whether the local accumulation of auxin was altered in the *hy5* mutants, we introduced the auxin-responsive marker, *DR5::GUS* (Ulmasov et al., 1997), into *hy5* mutants. *DR5::GUS* expression was strongly enhanced in the lateral roots of the *hy5* mutant compared to the WT plants (**Figure S3**), indicating that the loss of *HY5* function may alter the local accumulation of auxin to accelerate lateral root growth, thereby promoting RSA development.

### Expression Dynamics of HY5 in Response to Light

**Figure 2:**
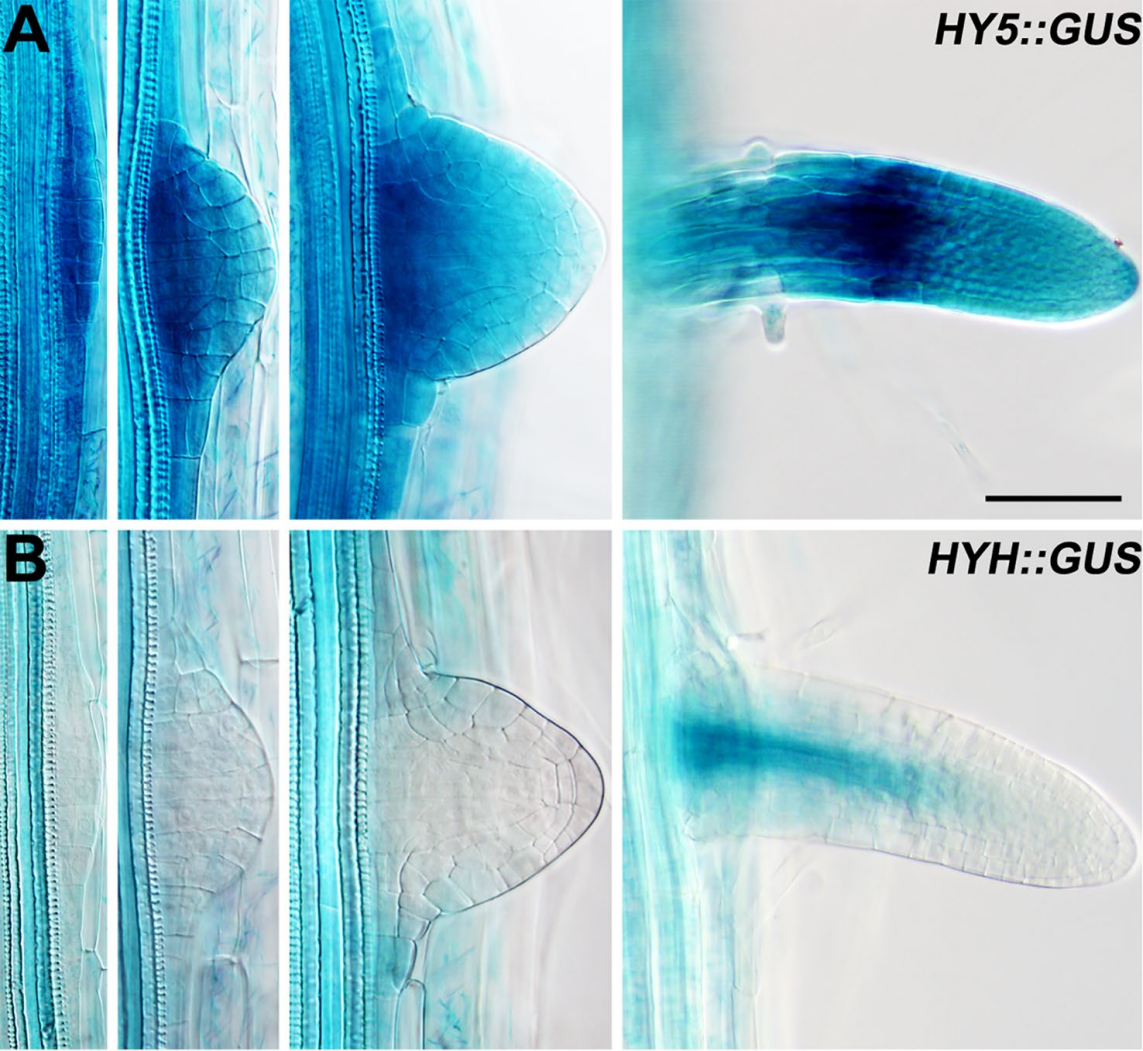
The expression patterns of *HY5* and *HYH* during lateral root development. **(A)** *HY5::GUS* roots. **(B)** *HYH::GUS* roots. The seedlings were grown in constant light for 8 days before the GUS staining. *Scale bar* represents 20 μm.

Lateral roots are a major component of the RSA (Smith and De Smet, 2012). As HY5 regulates RSA, we first examined the expression pattern of *HY5* during lateral root development using a *HY5* promoter reporter, *HY5::GUS*. This experiment revealed that *HY5* was abundantly expressed from the early stages of lateral root primordia development to the maturation of the lateral roots (**Figure 2**). *HY5::GUS* expression was predominately detected at the meristem and in the meristem-elongation transition zone of the mature lateral roots. In contrast, *HYH*, the *HY5* homolog, was only expressed in the vascular tissues of mature lateral roots. These data suggest that HY5 may be a crucial factor controlling RSA.

**Figure 3:**
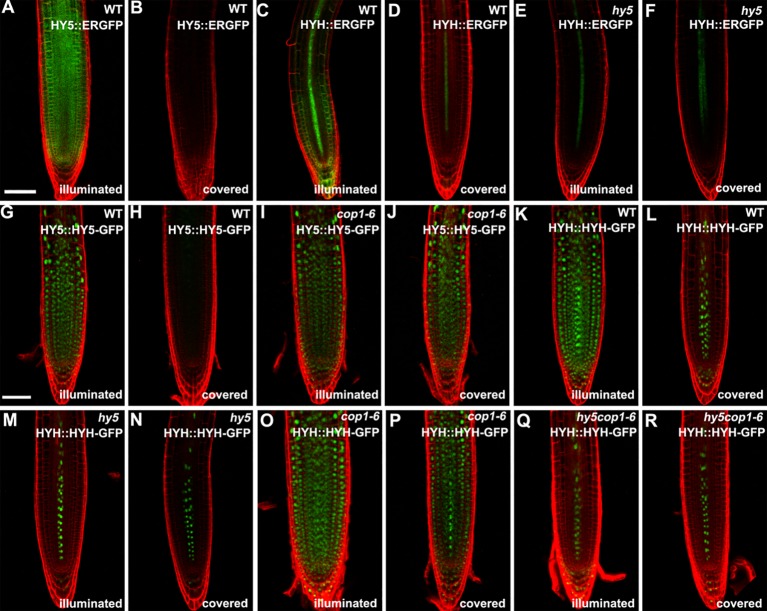
The expression patterns of *HY5* and *HYH* in response to darkness and light in the roots of seedlings grown in the root-covered system (RCS). **(A**, **B**, **G**, **H)** Expression of *HY5* promoter fusion **(A**, **B)** and protein fusion **(G**, **H)** in roots of wild-type (WT) seedlings in either illuminated or covered conditions as indicated. **(C**, **D**, **K**, **L)** Expression of the *HYH* promoter fusion **(C**, **D)** and protein fusion **(K**, **L)** in the roots of WT seedlings grown in either illuminated or covered conditions as indicated. **(E**, **F**, **M**, **N)** Expression of the *HYH* promoter fusion **(E**, **F)** and protein fusion **(M**, **N)** in the roots of *hy5* mutant grown in either illuminated or covered conditions as indicated. **(I**, **J)** Expression of the *HY5* protein fusion lines in roots of *cop1-6* mutant in either illuminated or covered conditions as indicated. **(O**–**R)** Expression of the *HYH* protein fusion lines in the roots of *cop1-6* mutant **(O**, **P)** and *hy5 cop1-6* mutants **(Q**, **R)** grown in either illuminated or covered conditions as indicated. *Scale bar* represents 20 μm.

Subsequently, we determined the expression of *HY5* and *HYH* in the roots in response to light. The transcript levels of *HY5* and *HYH* were measured in the roots of the illuminated and covered WT and *hy5* seedlings using qRT-PCR. As expected, the expression levels of *HY5* and *HYH* were significantly decreased in the foil-covered WT seedlings (WT-covered) compared to those exposed to light (WT-illuminated) (**Figure S4**). Consistently, the GFP signals in the *HY5* and *HYH* transcriptional GFP reporter lines expressing *HY5::ERGFP* or *HYH::ERGFP*, respectively, were dramatically enhanced by exposure to light (**Figures 3A–D**). The light-induced expression of *HYH* was abolished in the *hy5* mutant (**Figures 3C–F** and **Figure S4**), demonstrating that the transcriptional regulation of *HYH* is governed by HY5 in response to light.

### COP1 Mediates the Accumulation of HY5 Protein in Roots Directly Exposed to Light

The COP1-mediated degradation of proteins is known to be crucial for light-mediated growth and development (Osterlund et al., 2000; Holm et al., 2002). To determine whether COP1 mediates the accumulation of HY5 and HYH proteins in response to light in the roots, we next employed translational GFP reporters to investigate the levels of HY5 and HYH in plants of various genotypes with dark-grown or illuminated roots. The GFP signals of the WT plants expressing *HY5::HY5-GFP* or *HYH::HYH-GFP* were strongly decreased in plants with covered roots compared to the illuminated controls (**Figures 3G, H, K, L**), demonstrating that the expression of *HY5* and *HYH* in response to light occurs in a root-autonomous manner. Light did not appear to alter the GFP signals of the *cop1-6* mutant plants expressing *HY5::HY5-GFP* or *HYH::HYH-GFP* (**Figures 3I, J, O, P**). The *HYH::HYH-GFP* expression was found to be constantly inhibited in the *hy5* and *hy5 cop1-6* double mutants grown under the RCS (**Figures 3M–R**), indicating that the COP1-mediated proteasome degradation of HYH is dependent on HY5. Collectively, our data indicate that HY5 is a master coordinator of light-mediated root development.

### Expression Dynamics of HY5 in Response to Light Under the Soil-Grown Condition

**Figure 4:**
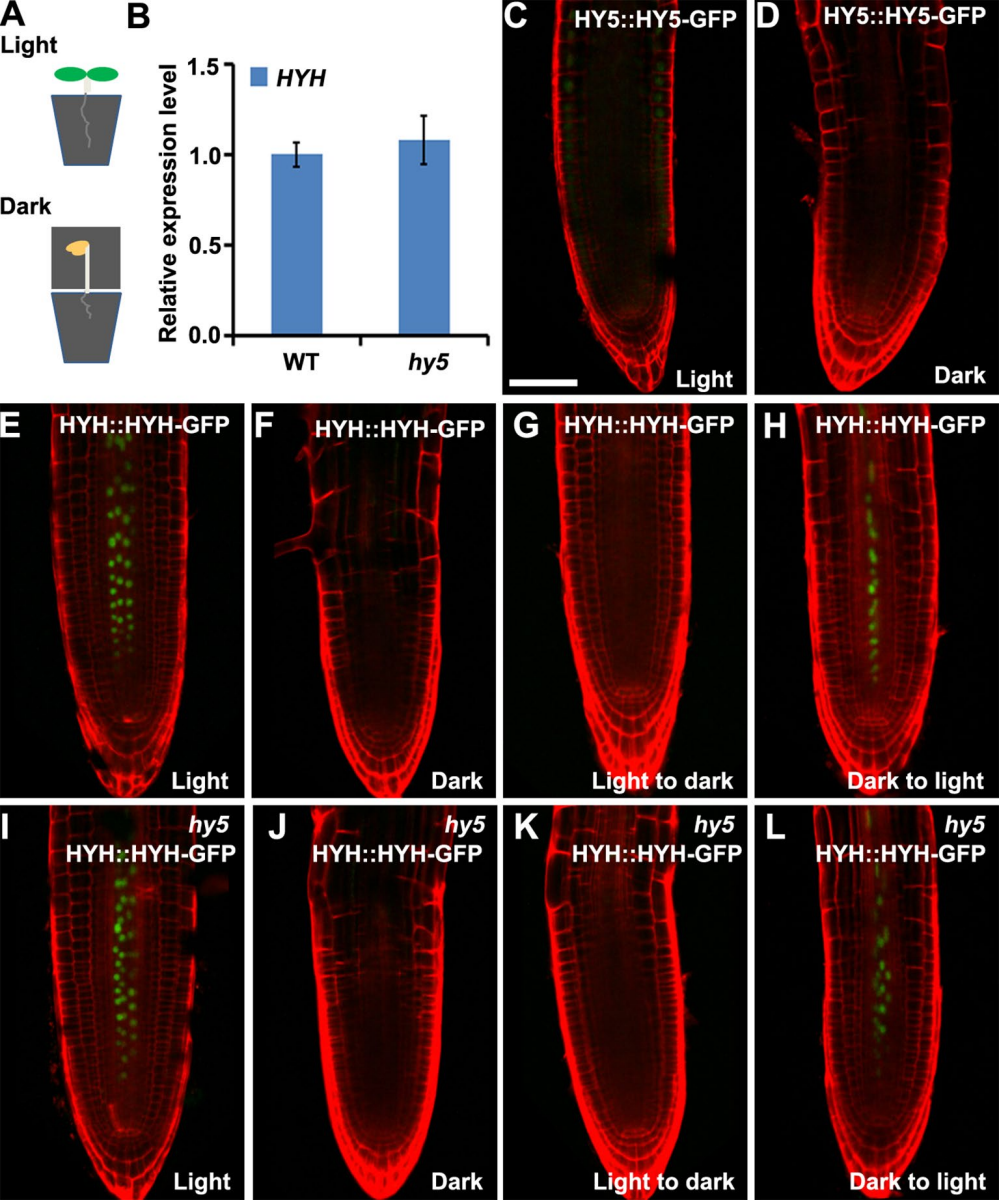
The expressions of HY5 and HYH in response to light in the soil. The expression levels of *HY5* and *HYH* in soil-grown seedlings in response to illumination of the shoot. **(A)** Illustration of the soil growing system used to perform the light treatments on the *Arabidopsis* seedlings. **(B)** qRT-PCR quantification of *HYH* transcripts in soil-grown wild-type (WT) and *hy5* seedlings when the shoots were exposed to light. *Error bars* represent the standard deviation (SD) of biological triplicates. *HYH* transcripts were normalized to the *EF1α* gene. **(C**, **D)** Expression of *HY5* protein fusion lines in the roots of soil-grown WT plants whose shoots were exposed to light **(C)** or darkness **(D)**. **(E**–**H)** Expression of *HYH* protein fusion lines in the roots of soil-grown WT plants whose shoots were subjected to light **(E)**, darkness **(F)**, or a light-to-dark **(G)** or dark-to-light **(H)** transition. **(I**–**L)** Expression of the *HYH* protein fusion lines in the roots of soil-grown *hy5* plants whose shoots were subjected to light **(I)**, darkness **(J)**, or a light-to-dark **(K)** or dark-to-light **(L)** transition. *Scale bar* represents 20 μm.

**Figure 5:**
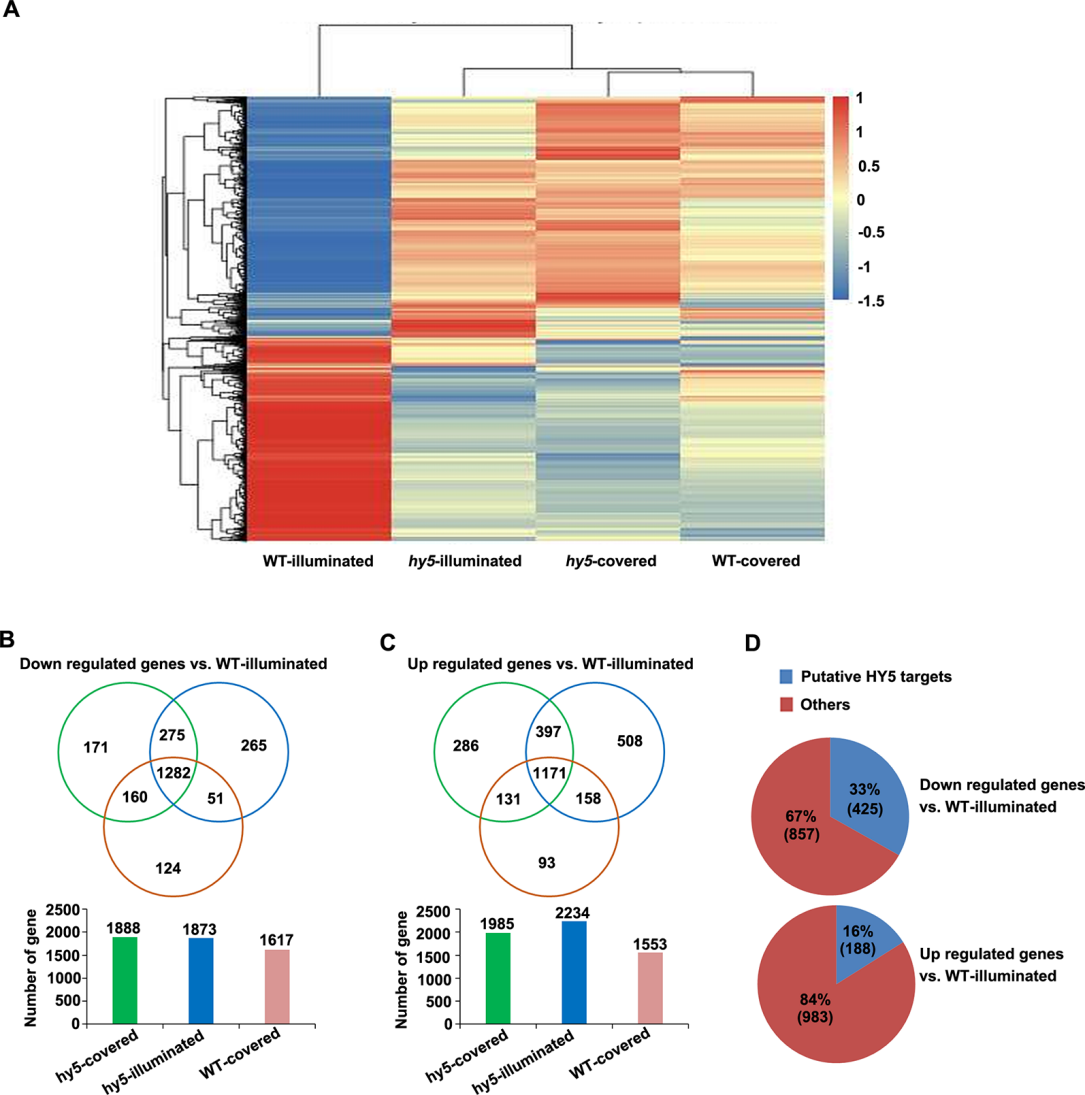
Analysis of the differentially expressed genes (DEGs) identified in the comparisons between the transcriptome of *hy5*-illuminated, *hy5*-covered, and wild-type (WT)-covered samples with those of WT-illuminated. **(A)** Overall clustering analysis and heat map of the four groups of root samples (WT-illuminated, *hy5*-illuminated, *hy5*-covered, and WT-covered). The gene expression values of each sample were averaged and *z*-transformed, resulting in scaled expression values between −1.5 and +1.5 for each gene. Hierarchical clustering was calculated using the hclust() function in R. **(B**, **C)** Venn diagram illustrating the number of unique (*non-overlapping circles*) and common (*overlapping circles*) upregulated and downregulated transcripts identified in *hy5*-illuminated, *hy5*-covered, and WT-covered compared to WT-illuminated. **(D)** Proportion of upregulated and downregulated transcripts that overlap with the list of HY5 targets.

*Arabidopsis* roots naturally grow in darkness in soil. To investigate whether HY5 plays a crucial role in soil-grown root development, we investigated the expression of *HY5::HY5-GFP* and *HYH::HYH-GFP* in plants whose shoots were subjected to light–dark transitions in soil-grown conditions. The seedlings were germinated on soil covered with a thin layer of sand to shield the roots from light. The shoot was either exposed to light or kept in darkness by covering with foil (**Figure 4A**). A qRT-PCR analysis revealed that the level of *HYH* expression was similar in the roots of soil-grown *hy5* and WT plants with light-exposed shoots (**Figure 4B**). Consistently, *HY5::HY5-GFP* accumulated at a relatively low level in the roots of soil-grown WT plants under shoot-illuminated and shoot-covered conditions (**Figures 4C, D**). *HYH::HYH-GFP* expression was much greater in the root vascular tissues of soil-grown seedlings when their shoots were exposed to light rather than darkness (**Figure 4E, F**) and in those transferred from darkness to light (**Figure 4H**). In contrast, almost no *HYH::HYH-GFP* expression was observed in the roots of plants whose shoots were either grown in darkness or transferred from light to dark (**Figures 4E–H**). No significant differences were observed in the *HYH::HYH-GFP* expression levels of the *hy5* and WT plants with shoots exposed to different light conditions (**Figures 5E–L**).

### Transcriptomic Analysis of HY5-Regulated Genes in Plants Grown Under RCS

To obtain new insights into the light-mediated regulation of RSA development by HY5 at a genome-wide level, we performed an RNA sequencing (RNA-Seq) study to compare the transcriptomic profiles of the light-exposed or dark-grown roots of 15-day-old WT and *hy5* seedlings. Differentially expressed genes (DEGs) were determined by comparing the transcriptomes of the illuminated or covered roots in *hy5* plants and by comparing the transcriptomes of the roots of WT plants grown in the two conditions. The clustered patterns of all DEGs (5,066 unique genes) were created based on their relative expression level values in all four samples (**Figure 5**). The results indicate that the gene expression patterns of the WT roots grown in darkness and the *hy5* roots grown in both illuminated and covered conditions contrasted with the expression pattern of the WT roots exposed to light.

Compared to the illuminated WT root sample (WT-illuminated), 1,985 genes, 2,234 genes, and 1,553 genes were upregulated in the *hy5* plants with covered roots (*hy5*-covered), the *hy5* roots exposed to light (*hy5*-illuminated), and the WT dark-grown roots (WT-covered), respectively, while 1,888 genes, 1,873 genes, and 1,617 genes were downregulated in these lines, respectively (**Figures 5B, C** and **Table S1**). We next investigated the overlapping DEGs in clusters of *hy5*-illuminated, *hy5*-covered, and WT-covered. One thousand two hundred eighty-two genes were downregulated and 1,171 genes were upregulated relative to their expression in WT plants with illuminated roots (WT-illuminated) (**Figures 5B, C** and **Table S1**).

Previous studies documented that the ACE element (ACGT) and G-box motif are the putative binding sites of HY5 (Lee et al., 2007). A comparative analysis of our data with the previous genome-wide microarray data of putative HY5 binding targets revealed that 425 of the 1,282 downregulated genes (33%) and 188 of the 1,171 upregulated genes (16%) are putative HY5 targets (**Figure 5D** and **Table S2**). In a previous summary of light signaling genes (Shikata et al., 2014), 29 of the downregulated genes identified here were shown to be involved in categories of COP/DET complex, such as *phyA-105 (SPA1)-related 3* (*SPA3*) and *SPA4*; PHY and Phot signaling, namely, *ROOT PHOTOTROPISM 2* and *RPT3/NON-PHOTOTROPIC HYPOCOTYL3* (*NPH3*); and COP1 substrates encoding genes including *HY5* and *BZS1/B-BOX DOMAIN PROTEIN 20* (*BBX20*). Among these 29 genes, 16 were putative HY5 targets (**Table S3**). Taken together, the HY5-targeted genes are enriched in light growth responses, which make a major contribution to root photomorphogenesis when grown under the RCS.

Phytochrome signaling induces genome-wide alternative splicing (AS) during de-etiolation to mediate light responses in *Arabidopsis* seedlings (Shikata et al., 2014). To reveal whether HY5-regulated RSA development is associated with AS changes, we explored four major AS events—exon skipping, intron retention, alternative 5′ splice sites, and alternative 3′ splice sites—in the transcriptome profiles of WT-illuminated, *hy5*-illuminated, *hy5*-covered, and WT-covered. Intron retention was found to be the most abundant AS event in each group, while exon skipping was the least common (**Figure S5** and **Table S4**). There were no differences among the frequency of these four AS events in either the individual samples or the overlapping clusters (**Figure S5**). Moreover, a low proportion of these AS events were accompanied by altered transcriptional expression levels (**Table S4**). Collectively, these data indicated that light-regulated AS might not be involved in the HY5-mediated regulationof RSA development in response to light.

### GO Functional Enrichments of HY5-Regulated Genes in Plants Grown Under RCS

**Figure 6:**
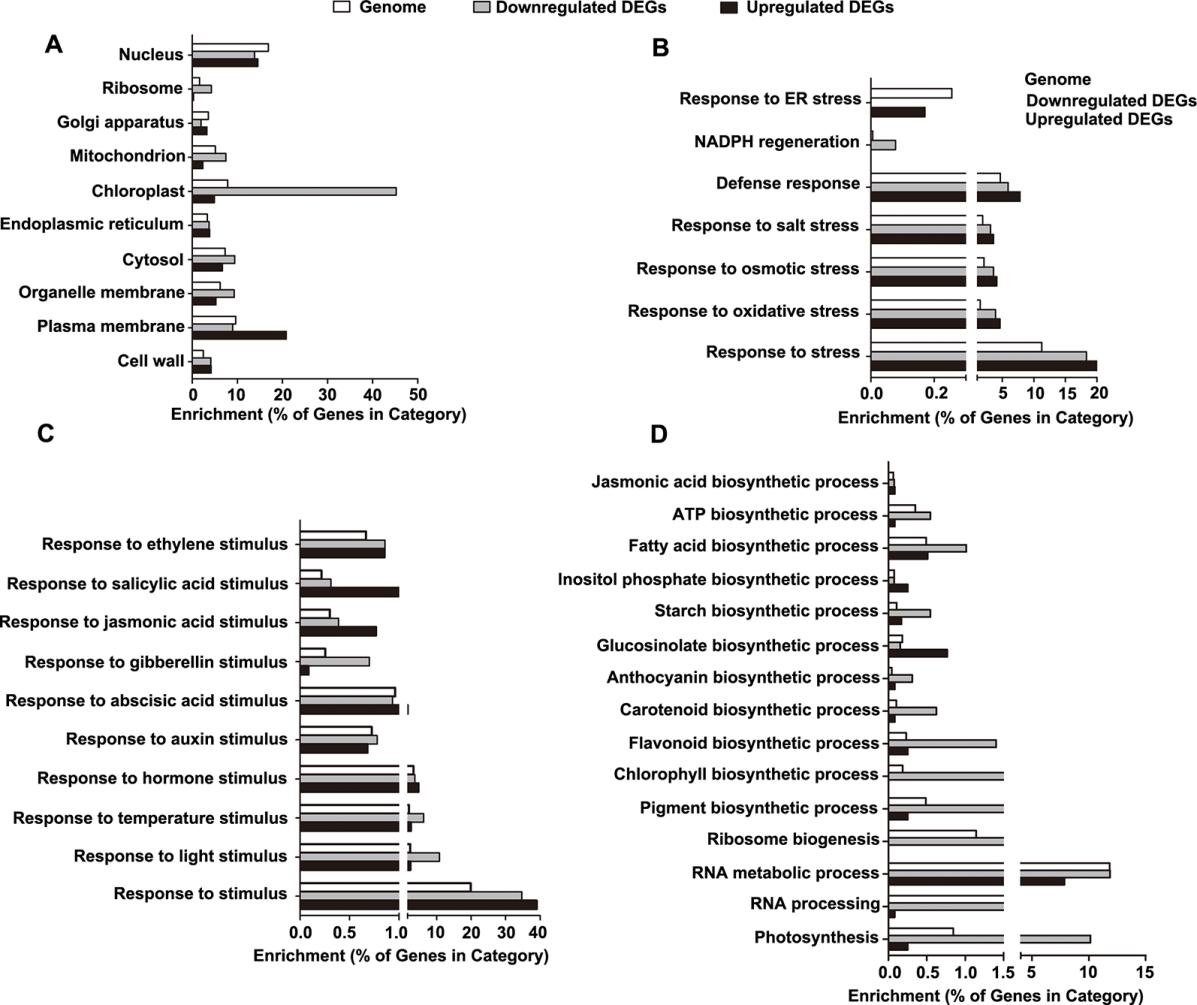
Gene ontology (GO) enrichment analysis of the differentially expressed genes (DEGs). **(A)** Categories of cellular component-related GO terms. **(B)** Categories of stress-related GO terms. **(C)** Categories of stimulus-related GO terms. **(D)** Categories of biosynthesis process-related GO terms.

To investigate the biological processes regulated by HY5, we categorized the common DEGs found in clusters of *hy5*-illuminated, *hy5*-covered, and WT-covered into GO terms. The processes were first grouped according to ontologies reflecting cellular components (CC) (**Figure 6A** and **Table S5**). The downregulated DEGs were mostly enriched in the chloroplast, organelle membrane, and ribosome categories, while the upregulated DEGs were enriched in the category of the plasma membrane (**Figure 6A** and **Table S5**).

Next, we determined the enrichment of the GO terms reflecting biological processes (BP), particularly those related to stress, stimuli, and biosynthetic processes. For stress-related GO terms, the categories of defense, salt stress, osmotic stress, NADPH regeneration, and oxidative stress were significantly overrepresented in the downregulated DEGs, while the category of endoplasmic reticulum (ER) stress was exclusively enriched in the upregulated DEGs (**Figure 6B** and **Table S5**). For stimulus-related GO terms, the categories of both environmental stimuli (light and temperature) and hormonal stimuli (auxin, abscisic acid, gibberellins, jasmonic acid, salicylic acid, and ethylene) were enriched in the downregulated DEGs (**Figure 6C** and **Table S5**). For biosynthetic process-related GO terms, the downregulated DEGs were enriched in the categories involved in the processes of ribosome biogenesis, RNA metabolic process, RNA processing, photosynthesis, and biosynthesis of jasmonic acid, ATP, fatty acids, inositol phosphate, starch, glucosinolate, anthocyanin, carotenoid, flavonoid, chlorophyll, and pigment (**Figure 6D** and **Table S5**).

### Transcriptomic Comparison of Plants Grown in the RCS With Those Grown in the D-Root System

Using a novel D-root system, in which the roots of the cultivated plants were grown in darkness while the shoot was in the light, Silva-Navas and colleagues revealed that light illumination inhibits root elongation (Silva-Navas et al., 2015; Silva-Navas et al., 2016). In a transcriptomic analysis, they identified 1,553 upregulated and 1,617 downregulated genes in the roots of seedlings with illuminated roots compared to the dark-grown roots (Silva-Navas et al., 2016). To compare the transcriptomes of plants grown in the RCS with those grown in the D-root system, we first compared the DEGs of the WT plants grown in the RCS with dark-grown or light-exposed roots to the DEGs identified for plants grown in the D-root system. In both systems, 575 genes were differentially regulated, among which, 114 common genes were upregulated and 316 common genes were downregulated in the roots grown in darkness compared to those under illumination (**Figure S6** and **Table S6**). A GO analysis suggested the response to salicylic acid, particularly the terpene biosynthetic and metabolic process, was significantly overrepresented in the upregulated transcripts, while in the downregulated genes, the significantly overrepresented categories were related to the response to abiotic stimulus, response to light stimulus, and photosynthesis (**Table S7**).

To further identify candidate HY5-related components involved in the responses of the root to its surrounding light and dark conditions, we further overlapped the DEGs of WT-covered, *hy5*-illuminated, and *hy5*-covered compared to WT-illuminated under RCS with that under D-roots (Silva-Navas et al., 2016). The DEGs between the roots of the dark-grown WT roots and the illuminated WT roots were compared to those of the *hy5* mutant under these two conditions. The expression patterns of 11 upregulated genes and eight downregulated genes in the WT comparison were abolished or partially compromised in the *hy5* mutant background (**Figure S6** and **Table S6**). This suggests that these genes might contribute to the HY5-mediated regulation of the root response to light. Accordingly, four of these 19 genes harbor putative HY5 binding sites in their promoter regions. It will be of interest to dissect the functions of these genes in follow-up work.

### HY5 Regulates the Root Response to Light by Mediating the Flavonol Content in the Roots

Silva-Navas et al. (2016) reported that the accumulation of flavonols in the root transition zone mediates the root response to light and therefore proposed that flavonols function as local positional signals. Interestingly, four of the flavonoid biosynthetic genes (*4CL3*, *TT7*/*F3'H*, *FLS*, and *CHS*), which are putative HY5 targets, were downregulated in the DEG clusters of *hy5*-illuminated, *hy5*-covered, and WT-covered in comparison to that of WT-illuminated (**Table S1**), indicating that flavonoid biosynthesis might be involved in the HY5 mediation regulation of RSA development in response to light. To test this hypothesis, the flavonol contents in the illuminated and dark-grown roots of the WT and *hy5* plants were determined using HPLC. The roots of the WT plants grown under light accumulated significantly higher levels of kaempferol and quercetin, the main flavonol derivatives, than WT-covered, *hy5*-illuminated, and *hy5*-covered (**Figure S7**), suggesting that illumination induces the accumulation of flavonols in a HY5-dependent manner. Next, we investigated whether the application of exogenous quercetin could revert the RSA phenotypes of the *hy5* mutant. The quercetin treatment decreased the primary root elongation, area of root extension, and number of lateral roots in both the WT and *hy5* roots, regardless of whether they were exposed to light or darkness (**Figures S8A–D**).

Flavonols regulate root growth in response to light by decreasing the accumulation of ROS in the root meristem (Silva-Navas et al., 2016). Since HY5 is indispensable for the accumulation of flavonols in response to light, we monitored the ROS levels in the roots of the *hy5* mutant. We found that the ROS levels were much higher in the roots of the dark-grown and illuminated *hy5* roots than those of the dark-grown and illuminated WT roots (**Figure S9**). Taken together, these results suggest that HY5 might regulate the root response to light by mediating the accumulation of flavonol in the roots.

## Discussion

### The RCS Is a Reliable Culture System That Can Mimic Soil Conditions

Traditionally, *Arabidopsis* seedlings were cultured in transparent Petri dishes in the laboratory, where both the shoots and roots are exposed to light. In nature, the roots of most terrestrial plants grow underground in darkness and grow away from light in a process known as negative phototropism. It has been reported that all photoreceptors are expressed in the roots to sense external light changes (Yokawa et al., 2014; Mo et al., 2015); therefore, many researchers have argued that investigating root development in transparent Petri dishes is inappropriate (Han et al., 2015; Mo et al., 2015). Recently, several improved cultural systems have been developed to study root development. In these systems, the shoots and roots are grown in separate light environments, which can be altered to explore the effects of light and darkness on the roots (Xu et al., 2013; Yokawa et al., 2013; Silva-Navas et al., 2015; Sakaguchi and Watanabe, 2017). Here, we developed an RCS, in which the roots can be grown in darkness by covering the Petri dish with foil. Our comparative analysis indicated that the RCS provides growth conditions similar to the soil environment; for example, the expression levels of *HY5* and *HYH* were similar between plants grown in the RCS and in the soil (**Figures 3** and **4**). Compared to the microtomography method, which uses X-ray CT scans to image the RSA (Lucas et al., 2011; Rellán-Álvarez et al., 2015), our RCS is more conventional and effective for use in the laboratory.

Using the RCS system, we revealed that the RSA was dramatically enhanced when the roots were covered with foil. Similar RSA phenotypes were observed in two recent reports (Silva-Navas et al., 2015; Van Gelderen et al., 2018a). This darkness-promoted root growth has been observed in other species; for example, Weller et al. reported that soil-grown pea (*Pisum sativum*) seedlings produced more lateral roots than seedlings grown in transparent plants with roots directly exposed to light (Weller et al., 2009). These data clearly show that our RCS is a useful and reliable culture approach for mimicking soil growth conditions.

### HY5 Is a Major Regulator of RSA Development

HY5 plays multifaceted roles during plant growth and development (Gangappa and Botto, 2016). Here, we demonstrated that the avoidance of direct light exposure could enhance the RSA in a HY5-dependent manner. The inhibition of RSA development by direct light exposure was not observed in the *hy5* mutants (**Figure 1**). Direct light exposure induced the transcriptional upregulation and protein accumulation of HY5 in the WT, which were decreased by darkness (**Figures 3** and **4**). This might explain the observation that the RSA of the *hy5* roots exposed to light was similar to that of the dark-grown WT plants (**Figure 1**). The inhibition of growth by HY5 was also previously observed for the hypocotyl and lateral roots (Oyama et al., 1997; Sibout et al., 2006). Moreover, the phytochrome-dependent accumulation of HY5 was found to regulate lateral root development when the shoots were exposed to far-red light (Van Gelderen et al., 2018a). In addition, light-grown pea seedlings deficient in *HY5* activity showed a phenotype similar to that of the dark-grown WT roots, although RSA development was significantly inhibited when the WT roots were exposed to light, suggesting that the mechanism by which HY5 inhibits growth might be conserved among species (Weller et al., 2009). A dramatic accumulation of HY5 occurred in the dark-grown *cop1-6* roots (**Figure 3**), suggesting that the accumulation of HY5 in dark-grown roots may be negatively regulated by COP1-mediated proteasome degradation (Osterlund et al., 2000). HY5 stability in early seedlings is also affected by the phosphorylation status in its COP1 binding domain (Hardtke et al., 2000; Osterlund et al., 2000). These evidences suggest that plants might utilize a similar process for the regulation of HY5 activity in different tissues.

### Possible Mechanisms by Which HY5 Regulates RSA

Our transcriptomic analysis revealed that over 2,000 genes were differentially expressed between roots grown in darkness and those exposed to light (**Figure 5**). Around 42% of DEGs (848 of the 2,021 DEGs) contained putative HY5 binding targets, highlighting the central role that HY5 may play in regulating RSA in response to light. These HY5-dependent genes were involved in various biological processes (**Figure 6**), suggesting that the mechanisms by which HY5 regulates RSA are complex.

Phytohormones are known to be important for root development (Zhao et al., 2015; Li et al., 2019b; Liu et al., 2019b); therefore, the modulation of hormone signaling may be one mechanism by which HY5 mediates RSA development. The stimulus categories containing the responses to plant hormones, including auxin, abscisic acid, gibberellins, jasmonic acid, salicylic acid, and ethylene, were all overrepresented in the HY5-dependent DEGs. All of these hormones, particularly auxin, have been well known in regulating root developments (Jiao et al., 2013; Pacifici et al., 2015; Li et al., 2017b; Lv et al., 2018). We also found that the loss of HY5 function increased the local auxin accumulation in the lateral roots (**Figure S3**).

HY5-mediated stress responses may also contribute to the regulation of RSA development. Genes involved in responses to stresses such as salt, osmosis, and oxidation were downregulated in roots grown in darkness compared to those exposed to light (**Figure 6**), suggesting that the defense response may be more active in the light-grown roots (Cui et al., 2019). A higher defense capacity is predicted to consume more energy, thereby reducing the energy available for growth (Bhosale et al., 2018; Li et al., 2019b). Our data suggest that HY5 might control the activation of the defense systems, which is consistent with the previous report that HY5 negatively regulates the plant responses to ER stress (Nawkar et al., 2017). Increasing light intensities evoke ER stress by affecting the protein-folding capacity of this organelle. Consequently, the unfolded protein response (UPR) in the ER is negatively controlled by HY5, which transcriptionally represses the UPR marker gene *BINDING PROTEIN3* (*BIP3*) by directly binding to its promoter (Nawkar et al., 2017). It was reasoned that the increased tolerance of the *hy5* mutant to the ER stress conditions was caused by the induction of the UPR marker genes (Nawkar et al., 2017). Although the upregulated genes were exclusively enriched in the response to ER stress category in our dataset, *BIP3* was only found to be upregulated in the illuminated *hy5* roots, with no difference in expression between the light-exposed WT roots compared to the dark-grown WT or *hy5* roots. We cannot rule out the involvement of the ER stress response in the RSA response to root illumination, but it may not be a primary contributor to the HY5-mediated regulation of root growth in response to light exposure.

As a central regulator in the response to light, HY5-regulated biosynthetic processes might also affect RSA development. The downregulated DEGs detected in the various comparisons with the illuminated WT roots are enriched in the categories related to the chloroplasts and photosynthesis. Root greening did not occur under our 15-day illumination; however, a previous study revealed that a long-term exposure to light (30 days) dramatically promotes chloroplast development and results in root greening in the WT, but not in the *hy5* mutant (Oyama et al., 1997). This demonstrates that cellular and subcellular changes occur as a result of the altered biosynthetic processes in light-exposed roots.

Further study is still needed to clarify which biological process is disturbed by light to cause the inhibition of RSA. The direct illumination of the root increases the expression of the flavonoid biosynthesis genes in a HY5-dependent manner (**Figure 6D** and **Table S1** and **5**), suggesting that the carotenoid and flavonoid biosynthesis pathways might contribute to the inhibition of RSA development. An uncharacterized carotenoid-derived molecule, apocarotenoid, was reported to function in a non-cell-autonomous manner in the establishment of the lateral root pattern and the regulation of lateral root formation (Van Norman et al., 2014). Flavonoids are ubiquitous plant secondary metabolites, which are involved in the regulation of basipetal auxin transport, thereby regulating root development (Winkel-Shirley, 2002; Buer et al., 2010; Li et al., 2019a). Defects in flavonoid biosynthesis result in a conditional increase in lateral root density (Buer et al., 2010). Until recently, flavonols were proposed to function as local signals, integrating the hormonal and ROS pathways to regulate root growth in response to light (Silva-Navas et al., 2016). In this study, we found that both darkness and the loss of *HY5* function decreased the flavonol content in the roots (**Figure S7**), while an exogenous supply of the major active flavonol intermediate quercetin restored the RSA phenotype of the *hy5* mutant regardless of the exposure of its root to illumination or darkness (**Figure S8**). This suggests that HY5 might regulate RSA in response to light by mediating flavonol production in the roots.

Lastly, ROS signaling might also be involved in the HY5-mediated regulation of RSA in response to light. Our transcriptome data showed that the genes involved in NADPH regeneration were enriched in the downregulated genes (**Figure 6B**; **Table S5**). NADPH is particularly important for ROS generation and metabolism (Noctor, 2006). Previous reports have also revealed that ROS plays a key role in regulating root growth and development (Jiao et al., 2013; Bai et al., 2014; Li et al., 2015). Additionally, a higher accumulation of ROS was previously observed in roots grown in darkness using a D-root system (Silva-Navas et al., 2015). We observed much higher ROS levels in the roots of the *hy5* mutant than in the WT root, whether they were grown in the darkness or exposed to light (**Figure S9**). These findings suggest that HY5 regulates RSA development in response to light by integrating flavonol production and ROS signaling.

Collectively, our results indicate that HY5 regulates RSA development through a complex network. Although we identified many putative HY5 targets that might influence RSA (**Table S2** and **6**), future studies should verify these targets and further dissect the mechanisms by which HY5 regulates RSA.

## Data Availability Statement

The raw sequence data reported in this paper have been deposited in the Genome Sequence Archive in BIG Data Center (Wang et al., 2017; Members, 2019), Beijing Institute of Genomics (BIG), Chinese Academy of Sciences, under accession numbers CRA001994 that are publicly accessible at https://bigd.big.ac.cn/gsa.

## Author Contributions

YHZ, CL, GW, and LZ: Conception and design, execution of experiment, analysis and interpretation of the data, and drafing of the article. HX, XS, JH, WZ, YZ, PH, K-XZ, XX, CW, XH, and CZ: Execution of experiment, analysis and interpretation of the data. ZH and WZ: Interpretation of the data and drafing of the article. XW, ZH, CW, HX, and WZ: Critical revision of the article for important intellectual content.

## Funding

This work was funded by the National Natural Science Foundation of China (31801210 to L.Z., 31701294 to C.L., and 31601191 to K.Z.), the Start-up Foundation of Hubei University of Medicine (2017QDJZR26, 2016QDJZR11, and 2016QDJZR14), the Open Research Fund of Key Laboratory of Medicinal Resources and Natural Pharmaceutical Chemistry Ministry of Education (2019005), the Foundation of Health Commission of Hubei Province (ZY2019Q004), the Fund for Key Laboratory Construction of Hubei Province (Grant No. WLSP201905), the Natural Science Foundation of the Jiangsu Higher Education Institutions of China (16KJB180002 to K.Z., in part), and the Priority Academic Program Development of Jiangsu Higher Education Institutions of China (in part).

## Conflict of Interest

The authors declare that the research was conducted in the absence of any commercial or financial relationships that could be construed as a potential conflict of interest.

## Supplementary Material

Figure S1 Illustration of a traditional culture system and the root-covered system. Representative photographs and corresponding schematic diagrams showing Arabidopsis seedlings grown on Petri dishes under TCS (A, B) and RCS (C). Scale bar represents 10 mm.

Figure S2 Root hair phenotypes of WT and hy5 roots in illuminated and dark-grown conditions grown using in the RCS. Representative images (A) and the quantification of root hair length (B) of WT and hy5 seedlings grown in illuminated and covered conditions under the RCS. Scale bar represents 1 mm. Error bars represent the SD of biological triplicates (n ≥ 20). Different letters indicate significantly different values at P < 0.05 (pairwise Student’s t-test).

Figure S3 DR5::GUS expression in the lateral roots of WT and hy5 seedlings. DR5::GUS expression in the early stage of lateral root growth (A and B) and in the mature lateral root (C and D). Scale bar represents 20 μm.

Figure S4 HY5 and HYH expression levels in the roots of WT and hy5 plants grown in illuminated and covered conditions in the RCS. qRT-PCR quantification of HY5 and HYH expression. Error bars represent the standard deviation (SD) of biological triplicates. HY5 and HYH transcripts were normalized to the EF1α gene. **P < 0.01, pairwise Student’s t-test.

Figure S5 Analysis of alternative splicing events in differentially expressed genes. Four types of splicing events were investigated from the transcripts of WT-illuminated, WT-covered, hy5-illuminated, and hy5- covered, respectively.

Figure S6 Transcriptomic comparison of plants grown in the RCS with those grown in the D-root system. (A) Venn diagram illustrating the overall overlaps of the DEGs between the illuminated and the dark-grown WT roots grown in the RCS (this study) and the same comparison in the D-root system (Silva‐Navas et at., 2015). (B and C) Venn diagram illustrating the overlap of upregulated DEGs (B) and downregulated DEGs (C) in the dark-grown versus illuminated WT roots. (D) Venn diagram illustrating the overlap of light-repressed genes (upregulated genes compared to the illuminated WT roots) in different clusters. (E) Venn diagram illustrating the overlap of light-induced genes (downregulated genes compared with illuminated WT roots) in different clusters.

Figure S7 The determination of flavonols in the roots of the WT and hy5 roots grown in illuminated and dark conditions. (A-B) An HPLC chromatogram of the flavonols in WT covered (A) and hy5 illuminated (B) compared to WT illuminated. (C-D) An HPLC chromatogram of the flavonols in hy5 covered compared to WT covered (C) and hy5 illuminated (D), respectively.

Figure S8 Flavonols regulate the root system architecture of plants grown under the RCS. (A) Representative pictures of the RSA phenotypes of 12-d-old WT and hy5 plants grown in 1/2 MS medium with a mock control or 50 μM quercetin. Scale bar represents 10 mm. (B-E) Quantification of the RSA phenotypes of 12-d-old WT and hy5 plants grown in 1/2 MS medium with a mock control or 50 μM quercetin under the indicated light conditions, including the primary root lengths (B), root extension area (C), lateral root number (D), and lateral root angle (E). Error bars represent the SD of biological triplicates (n ≥ 6). Different letters indicate significantly different values at P < 0.05 (pairwise Student’s t-test).

Figure S9 The ROS levels in the roots of WT and hy5 plants grown in illuminated or covered conditions. Fluorescein diacetate staining was used to visualize ROS. Scale bar represents 50 μm.

Table S1 Upregulated and downregulated genes in dark-grown hy5 and WT roots and illuminated hy5 roots compared to the illuminated WT roots.

Table S2 Putative HY5 target genes identified in the clusters of upregulated and downregulated differentially expressed genes (DEGs), respectively.

Table S3 Genes found to overlap in a comparison with a previous summary of light signaling genes reported by Shikata et al. (2014).

Table S4 Alternative splicing (AS) events analysis of DEGs.

Table S5 Gene Ontology (GO) enrichments of DEGs.

Table S6 Overlap of genes in the DEGs of dark-grown WT roots grown under the RCS and those grown in the D-root system.

Table S7 GO enrichment analysis in common regulated genes related to HY5.
