# Supplementary figures and images for: HY5 Contributes to Light-Regulated Root System Architecture Under a Root-Covered Culture System
Source: Front Plant Sci. 2019 Nov 28;10:1490. doi: 10.3389/fpls.2019.01490 (PMC6892842; doi:10.3389/fpls.2019.01490)

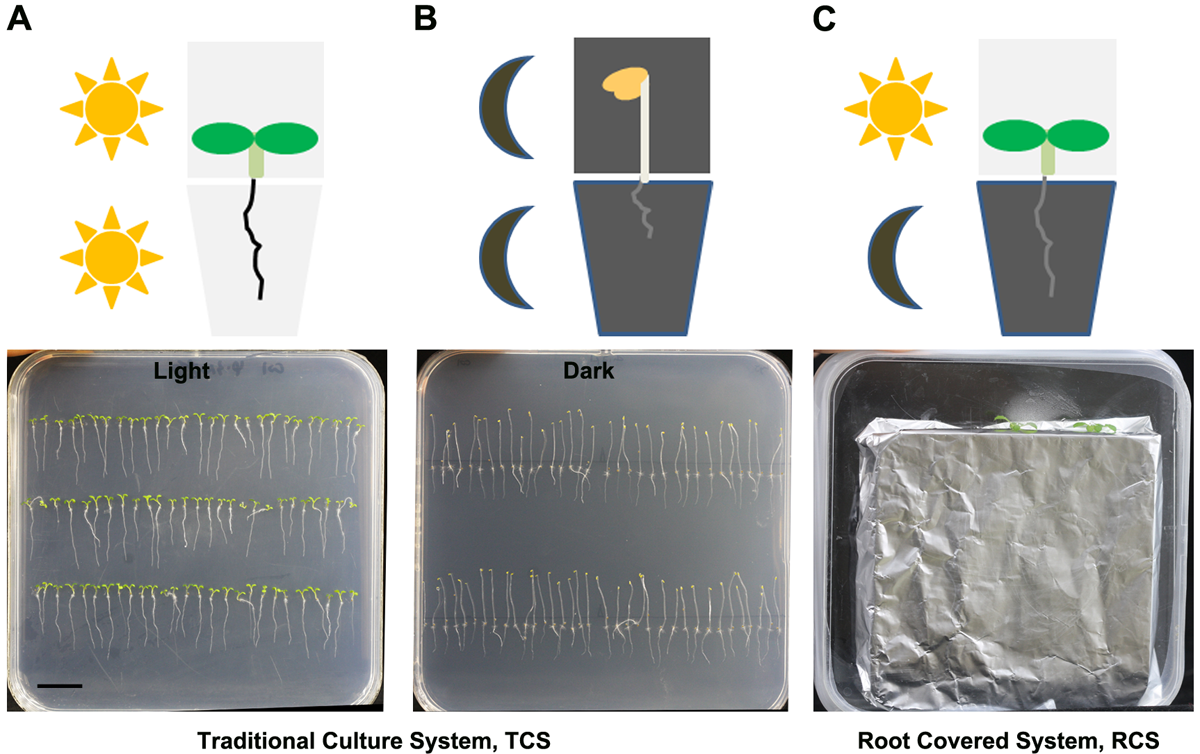

Supplement: Figure S1 — Illustration of a traditional culture system and the root-covered system. Representative photographs and corresponding schematic diagrams showing Arabidopsis seedlings grown on Petri dishes under TCS (A, B) and RCS (C). Scale bar represents 10 mm. [file Image_1.tif]

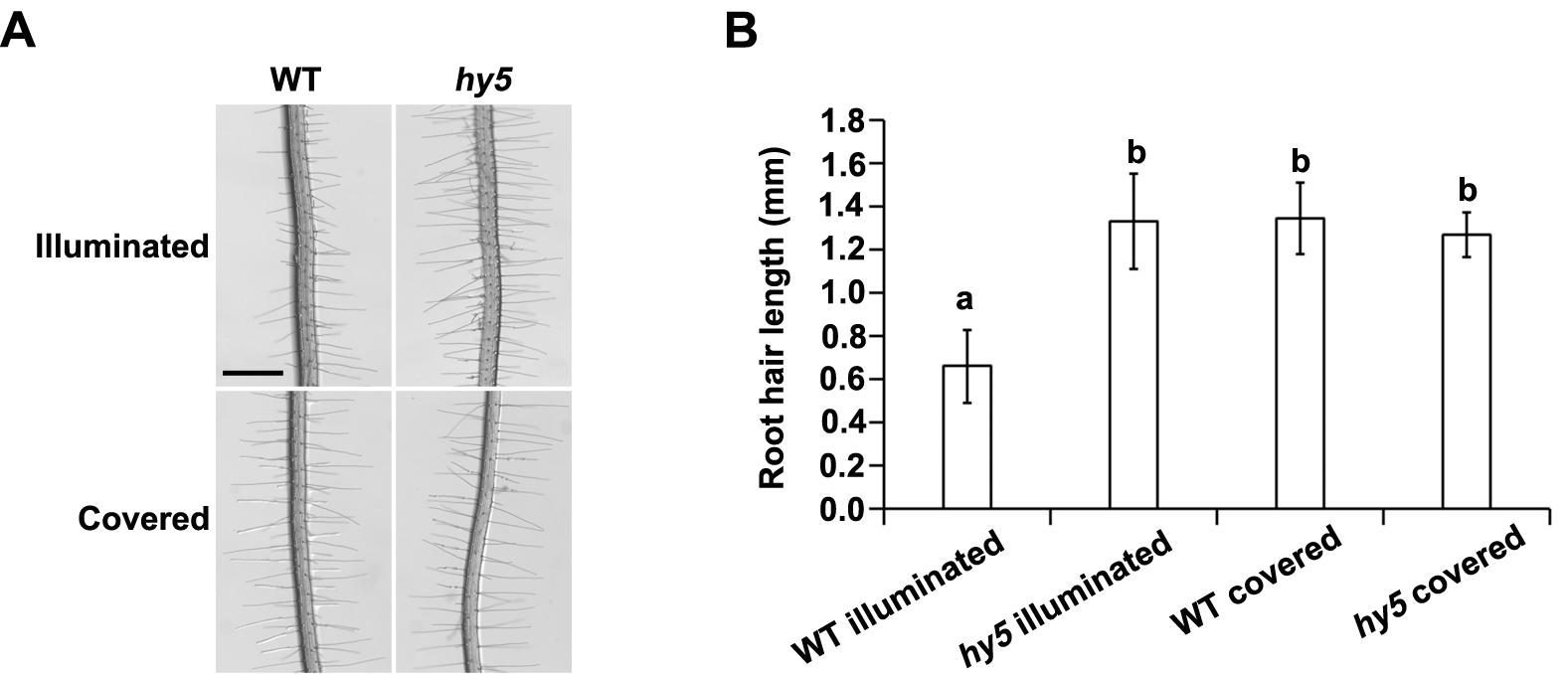

Supplement: Figure S2 — Root hair phenotypes of WT and hy5 roots in illuminated and dark-grown conditions grown using in the RCS. Representative images (A) and the quantification of root hair length (B) of WT and hy5 seedlings grown in illuminated and covered conditions under the RCS. Scale bar represents 1 mm. Error bars represent the SD of biological triplicates (n ≥ 20). Different letters indicate significantly different values at P < 0.05 (pairwise Student’s t-test). [file Image_2.tif]

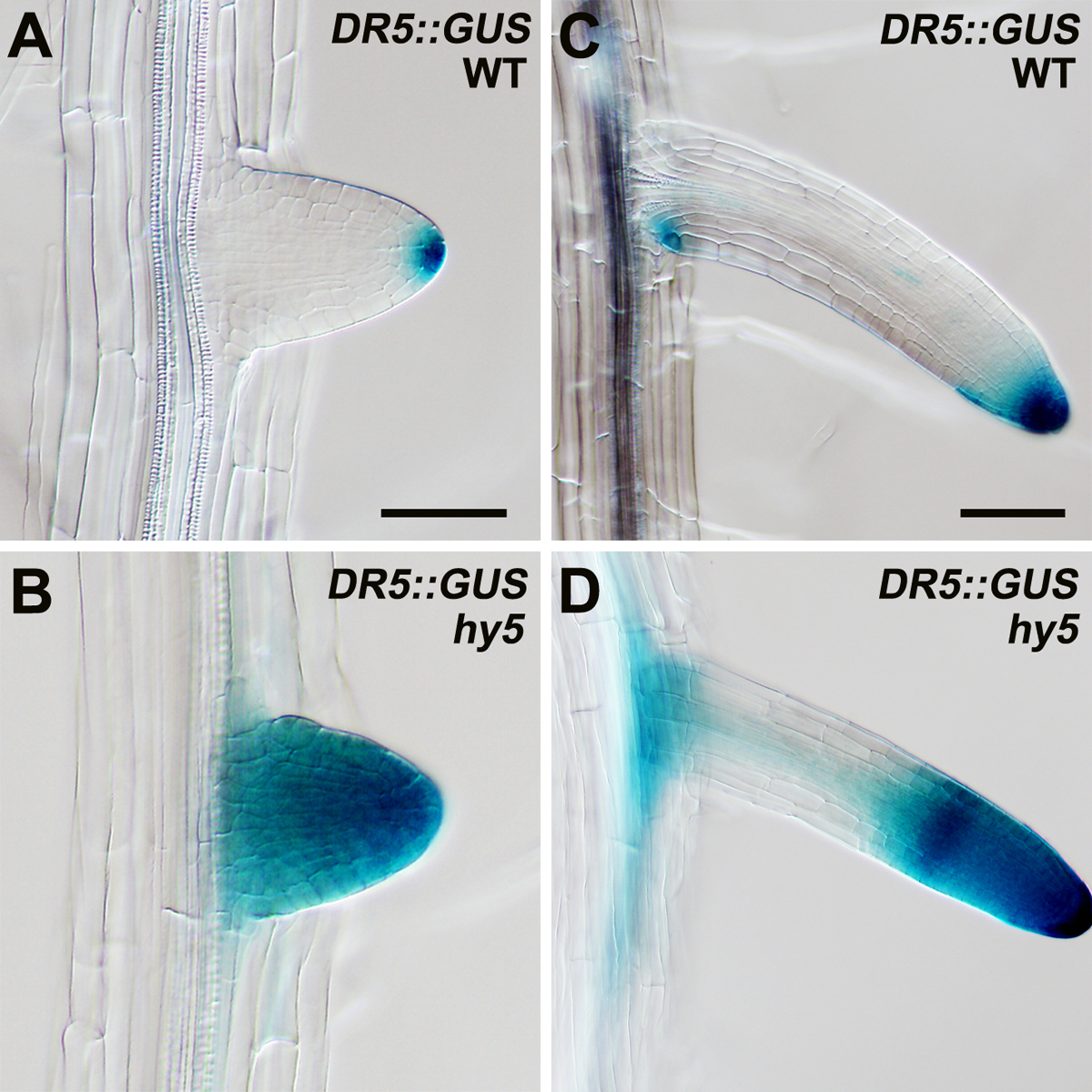

Supplement: Figure S3 — DR5::GUS expression in the lateral roots of WT and hy5 seedlings. DR5::GUS expression in the early stage of lateral root growth (A and B) and in the mature lateral root (C and D). Scale bar represents 20 μm. [file Image_3.tif]

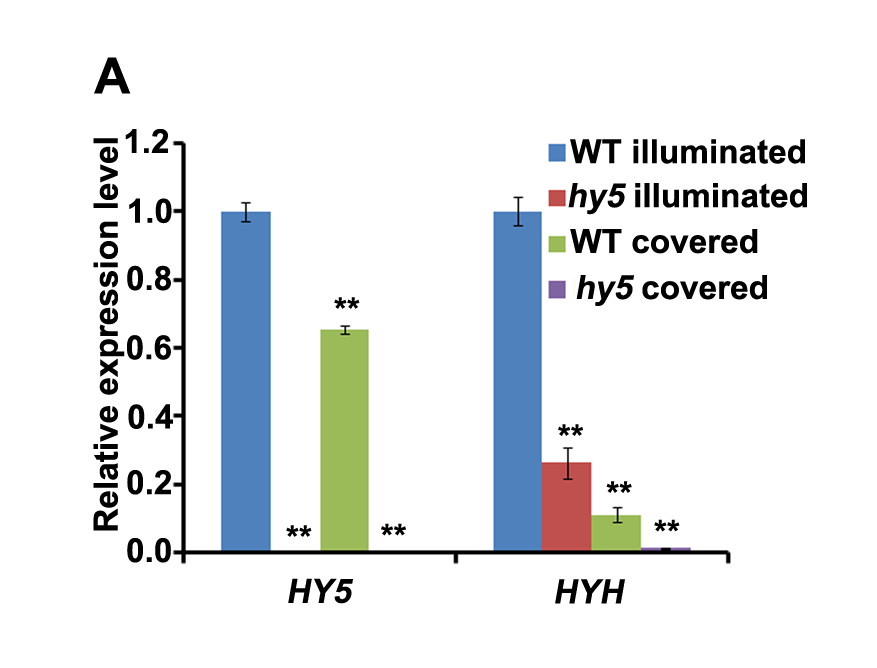

Supplement: Figure S4 — HY5 and HYH expression levels in the roots of WT and hy5 plants grown in illuminated and covered conditions in the RCS. qRT-PCR quantification of HY5 and HYH expression. Error bars represent the standard deviation (SD) of biological triplicates. HY5 and HYH transcripts were normalized to the EF1α gene. **P < 0.01, pairwise Student’s t-test. [file Image_4.tif]

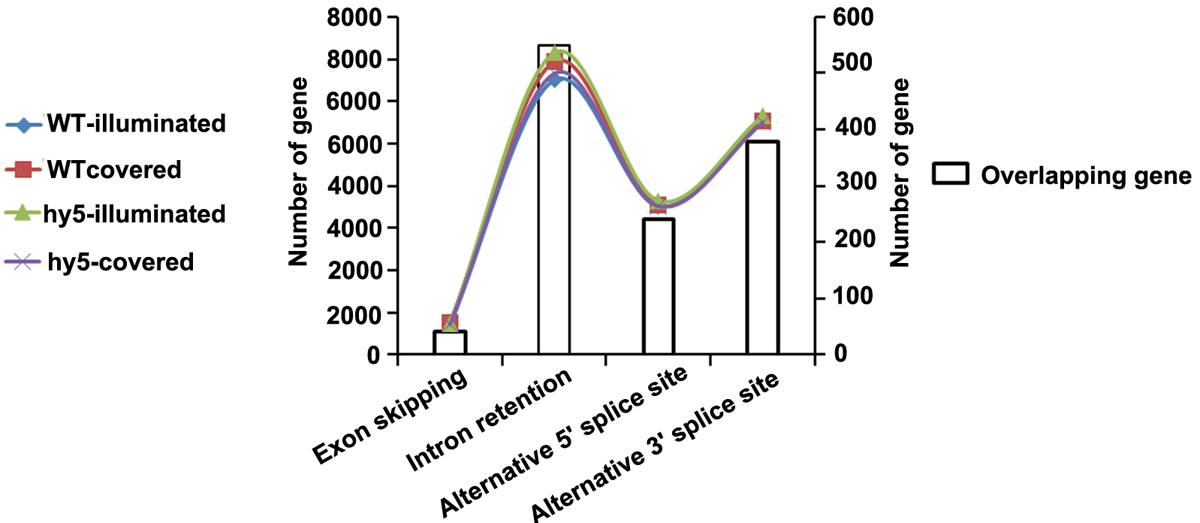

Supplement: Figure S5 — Analysis of alternative splicing events in differentially expressed genes. Four types of splicing events were investigated from the transcripts of WT-illuminated, WT-covered, hy5-illuminated, and hy5- covered, respectively. [file Image_5.tif]

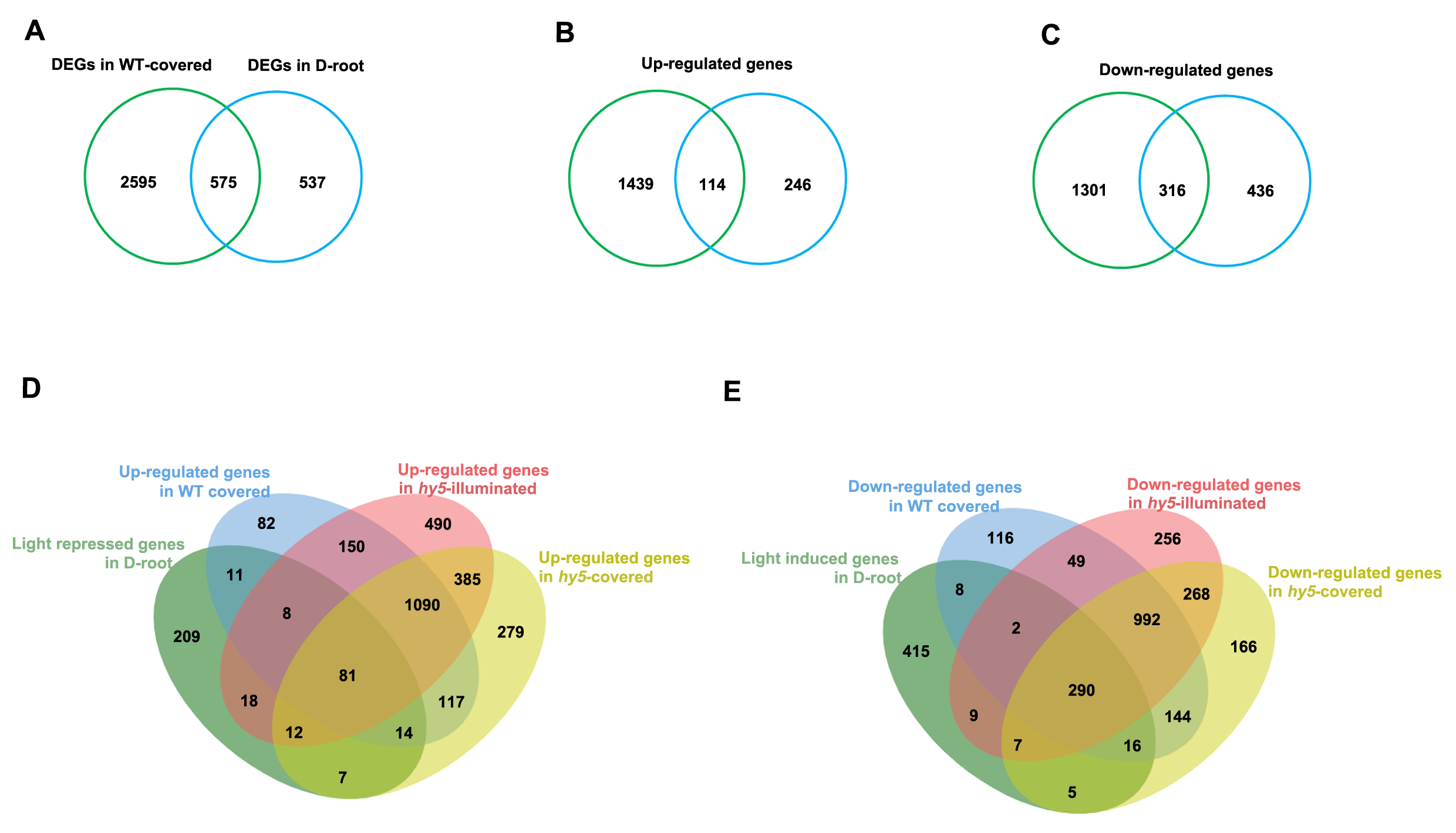

Supplement: Figure S6 — Transcriptomic comparison of plants grown in the RCS with those grown in the D-root system. (A) Venn diagram illustrating the overall overlaps of the DEGs between the illuminated and the dark-grown WT roots grown in the RCS (this study) and the same comparison in the D-root system (Silva‐Navas et at., 2015). (B and C) Venn diagram illustrating the overlap of upregulated DEGs (B) and downregulated DEGs (C) in the dark-grown versus illuminated WT roots. (D) Venn diagram illustrating the overlap of light-repressed genes (upregulated genes compared to the illuminated WT roots) in different clusters. (E) Venn diagram illustrating the overlap of light-induced genes (downregulated genes compared with illuminated WT roots) in different clusters. [file Image_6.tif]

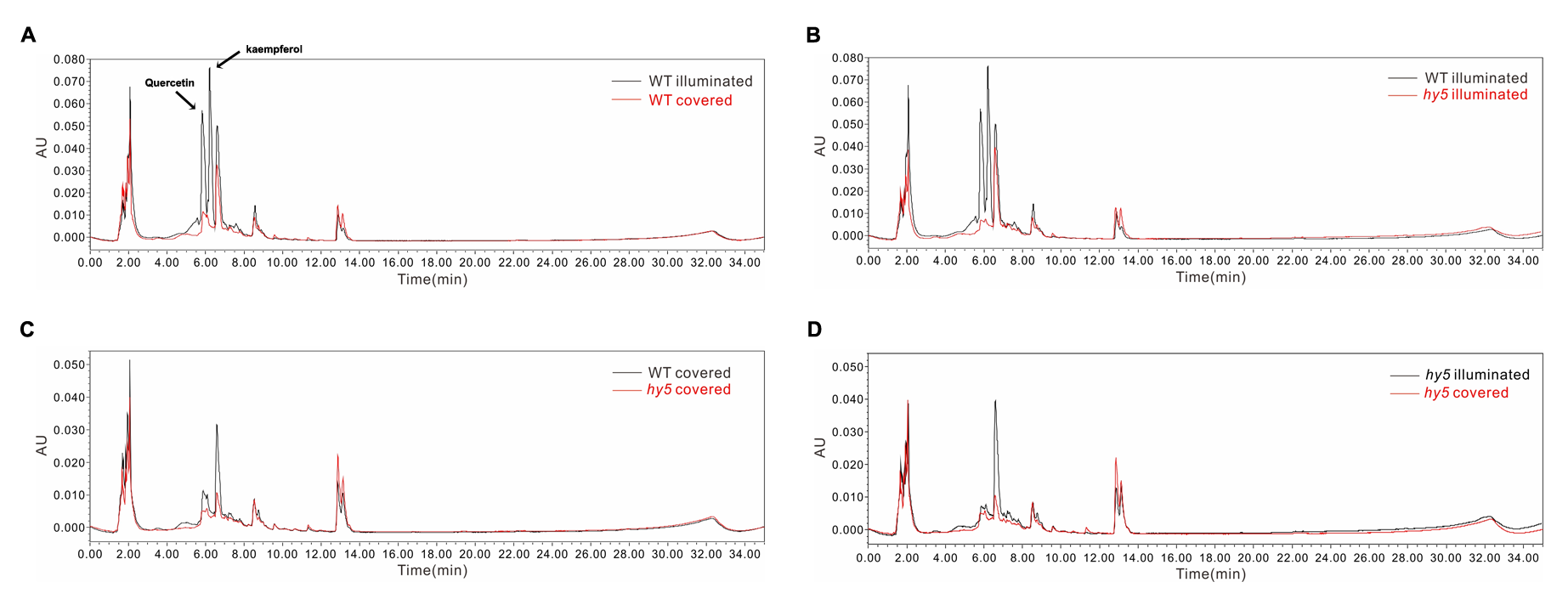

Supplement: Figure S7 — The determination of flavonols in the roots of the WT and hy5 roots grown in illuminated and dark conditions. (A-B) An HPLC chromatogram of the flavonols in WT covered (A) and hy5 illuminated (B) compared to WT illuminated. (C-D) An HPLC chromatogram of the flavonols in hy5 covered compared to WT covered (C) and hy5 illuminated (D), respectively. [file Image_7.tif]

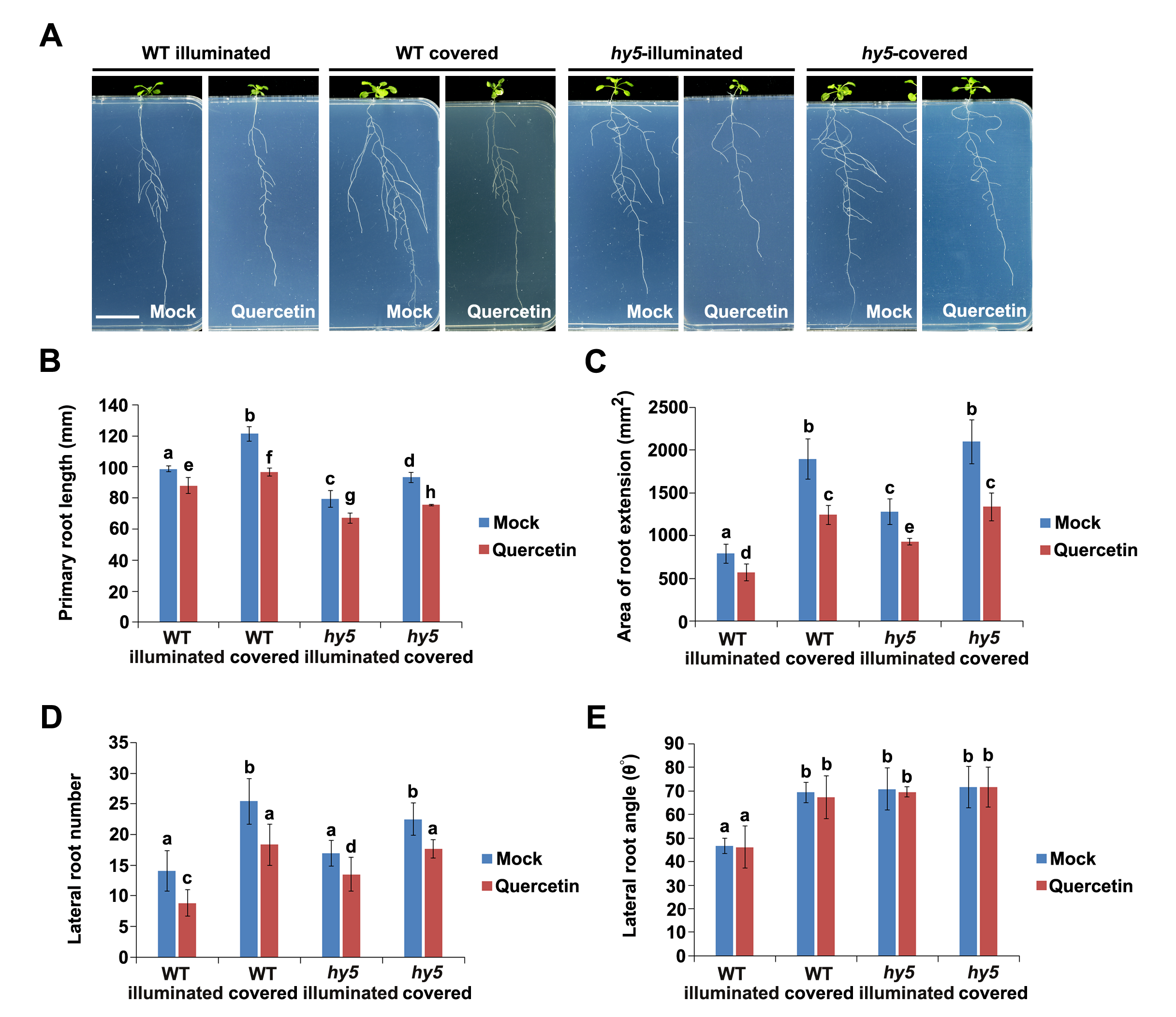

Supplement: Figure S8 — Flavonols regulate the root system architecture of plants grown under the RCS. (A) Representative pictures of the RSA phenotypes of 12-d-old WT and hy5 plants grown in 1/2 MS medium with a mock control or 50 μM quercetin. Scale bar represents 10 mm. (B-E) Quantification of the RSA phenotypes of 12-d-old WT and hy5 plants grown in 1/2 MS medium with a mock control or 50 μM quercetin under the indicated light conditions, including the primary root lengths (B), root extension area (C), lateral root number (D), and lateral root angle (E). Error bars represent the SD of biological triplicates (n ≥ 6). Different letters indicate significantly different values at P < 0.05 (pairwise Student’s t-test). [file Image_8.tif]

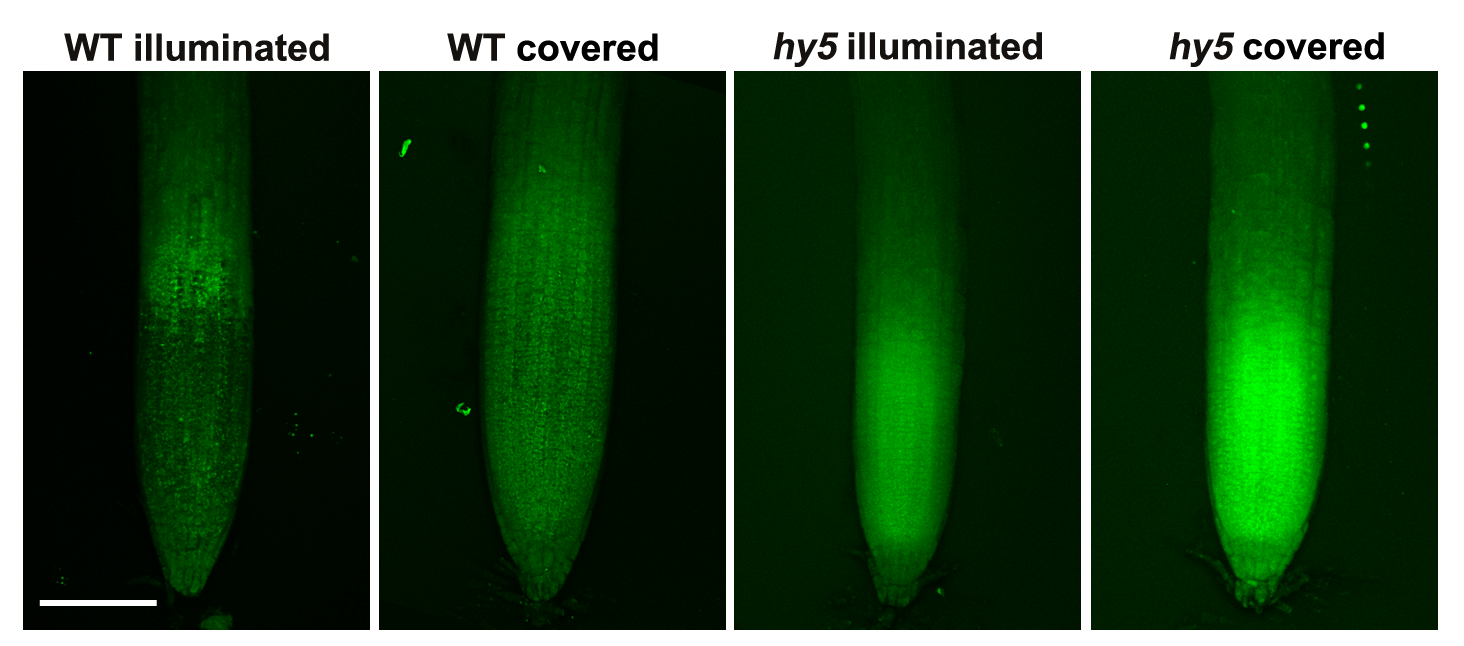

Supplement: Figure S9 — The ROS levels in the roots of WT and hy5 plants grown in illuminated or covered conditions. Fluorescein diacetate staining was used to visualize ROS. Scale bar represents 50 μm. [file Image_9.tif]
